# Movement Behavior of the Pine Needle Gall Midge (Diptera: Cecidomyiidae)

**DOI:** 10.1093/jisesa/ieaa121

**Published:** 2020-10-31

**Authors:** Huanxiu Liu, Chunhua Duan, Yukun Qi, Lili Ren, Haiwei Wu

**Affiliations:** 1 Shandong Academy of Forestry, Jinan, Shandong, P. R. China; 2 Beijing Forestry University, Beijing, P. R. China

**Keywords:** pine needle gall midge, flight capacity, tethered flight, temperature, age

## Abstract

The movement behavior of the pine needle gall midge (*Thecodiplosis japonensis* Uchida Et Inouye (Diptera: Cecidomyiidae)), an invasive species in China, was determined by using a tethered flight technique and digital videography in the laboratory. The flight distance, duration, and speed of females were compared at different ages (2–10 h) and ambient temperatures (17, 21, 26, and 30°C). Female flight distance and duration at 26°C were significantly greater than those at 17°C and 21°C. The age of *T. japonensis* did not significantly affect the three flight characteristics. For females at 2–10 h of age at 26°C and 70% RH, the maximum flight distance was 667.59 m; the longest flight time was 6,222.34 s; and the fastest flight speed was 0.44 m·s^−1^. For larvae wetted with water, the highest jump was 5.7 cm; the longest jump was 9.6 cm; and the greatest distance moved in 5 min was 27.13 cm, which showed that the active dispersal potential of larvae was very low.

Dispersal is a fundamental behavior in the life history and ecology of insects; it is important in avoiding natural enemies, searching for food, securing mates, and finding suitable habitat. Dispersal is also of central importance to population biology, behavioral ecology, and conservation (Rudd and McEvoy 1996). Therefore, insect dispersal has a key role in predicting population spread, improving eradication surveys, and successfully managing of destructive pest populations (Mazzi and Dorn 2012, Rafter et al. 2018). In addition, an understanding of the dispersal behavior of natural enemies can assist in developing effective augmentative release strategies and in assessing the spread and potential nontarget effects of an introduced natural enemy (Smith 1996, Orr et al. 2000). Dispersal (i.e., the spatial redistribution of populations) and movement (i.e., the spatial behavior of individuals) are closely related, because the random movement of individuals, in part, underlies the redistribution of a population in space (Turchin 1998, Nathan et al. 2008, Schellhorn et al. 2014). Insects move by walking or flying, and their movements are usually influenced by many intrinsic (physiological) and extrinsic (environmental) factors, such as temperature, wind, and photoperiod (Taylor 1963, Walters and Dixon 1984, McManus 1988, Roderick and Caldwell 1992, Nathan et al. 2008, Baguette et al. 2014).

In this study, the focus was on the movement behavior of the pine needle gall midge (*Thecodiplosis japonensis*). The pine needle gall midge is native to Japan and has been one of the most important pest of *Pinus densiflora* Siebold & Zucc. and *P. thunbergii* Parl. in Korea since the 1920s (Luo 1993, Park and Chung 2006). In 2016, the pine needle gall midge was first recorded in Huangdao, China, and found to attack three species of pine in the field: *P. thunbergii*, *P. densiflora*, and *Pinus tabuliformis* Carrière. In Korea, the pine needle gall midge has one generation per year. The larvae overwinter and then pupate in the soil in early May, with adults beginning to emerge at the end of May. Adults have a short life span of approximately 1 d, and therefore mate soon after emergence. After mating, females lay eggs between a pair of developing needles of the current-year shoots, with eggs hatching approximately 1 wk later. The newly hatched larvae creep to the leaf sheath and feed at the base of needles where they begin to form galls (Nam and Choi 2014). The growth of attacked needle pairs slow down substantially beginning in early July, in contrast to needle pairs that are not attacked. In winter, the attacked needles gradually wither and drop prematurely. Losses are appreciable after 2 and 3 yr of infestation, and seriously infested trees (more than 50% of needles on the current-year shoots are attacked) will wither and die (Soné 1986).

According to available data, the dispersion of the pine needle gall midge is very rapid, and the damage caused is very heavy. The pine needle gall midge was introduced into Korea from Japan in the 1920s and its spread has been described since 1929 (Choi et al. 2011). By 1960, the pine needle gall midge was found throughout Korea. In 1990, the distribution expanded into Cheju Island off the southern coast of South Korea (CABI 2019). Since 1972, approximately 300,000–400,000 ha of *P. thunbergii* forest have been damaged each year by *T. japonensis* in Korea (Park et al. 1985, Lee and Lee 1994). However, the flight capabilities of the pine needle gall midge have not been quantified. To tag adults and determine their flight range and dispersal, a technique was developed in South Korea in which the adults and larvae were labeled with radioactive phosphorus and calcium; however, the experiment failed (Kwon et al. 1978). Air speed is one factor that clearly affects the dispersal of pine needle gall midge adults. Ko and Lee (1975) found that few of the midges initiate flight when wind speeds are greater than that of a light breeze (2.8 m·s^−1^). Those insects already in flight are blown in the direction of the wind, whereas there is little or no flight in a moderate breeze (6.1 m·s^−1^).

*Pinus thunbergii*, *P. densiflora*, and *P. tabuliformis*, the three main hosts of the pine needle gall midge in the field, are widely cultivated for timber production and landscape use in China (Li et al. 2015, Ren et al. 2018). Although the pine needle gall midge is currently recorded only in the Huangdao area, it undoubtedly poses a serious threat to pine forest in China. Thus, determining the active dispersion ability of the pine needle gall midge is essential to develop control strategies and to identify new areas of potential invasion. Flying and crawling are the primary modes of movement in the life history of insects. Therefore, the current study was designed to answer the following general questions: 1) How long can *T. japonensis* fly? 2) What is the capacity of *T. japonensis* larvae for movement? 3) What are the effects of age and ambient temperature on flight behavior?

## Materials and Methods

### Insects

Newly emerged adults were collected by using five emergence traps. The emergence traps were composed of an opaque plastic cage (40 × 40 × 40 cm) and a white transparent packing box (volume, 60 ml; height, 100 cm; width, 4.2 cm). In a seriously damaged pure stand of *P. thunbergii* in Huangdao District, Qingdao City, Shandong province, China (35°58′14″N, 120°11′28″E), in late-V-19, soil (30 cm × 30 cm, to a depth of 10 cm) in which the pine needle gall midge pupated was collected in a plastic bag. The soil was spread evenly on the bottom of a cage in an insect rearing room at 25 ± 1.3°C and 45 ± 10% RH under 16:8 (L:D) h photoperiod. An opaque plastic cover that had a hole (diameter, 2 cm) in the center sealed the top of the cage. A white transparent packing box (volume, 120 ml; height, 10 cm; width, 4.2 cm) was used to cover the hole and collect the newly emerged adults. When an adult flew into the box, the box was replaced, and thus, each box contained one adult.

### Longevity of Adults

The newly emerged adults were kept individually in the white transparent packing boxes, and their survival was observed every 8 h in the field where they emerged from the soil. A midge was considered dead when none of its appendages moved after each was touched with a brush. The dead adults were sexed under a microscope (the females have a medium-length ovipositor of approximately 0.5 mm). The ambient temperature was 23–27°C, and the RH was 50–82%. No food or water was supplied during the tests. Within 5 d, 167 males and 100 females emerged. Thirty males and 30 females were randomly selected, and their time of death was recorded on the basis of hourly observation.

### Determination of Flight Characteristics

#### Pretreatment of adults

With a pair of tweezers, a cotton ball impregnated with anhydrous ether was inserted into a white transparent packing box with an adult. The cotton ball was removed when the adult was anesthetized (no appendage movement, approximately 15–20 s). An anesthetized adult was quickly placed on a piece of white filter paper, and the legs, wings, and abdomen were adjusted with pointed tweezers to expose its pronotum. Females were selected under a stereoscopic for use in the flight mill assay.

#### Flight mill assay

**Fig. 1.**
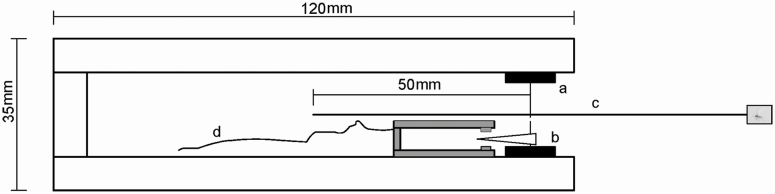
Schematic of the flight mill apparatus used to test the flight capacity of the pine needle gall midge (*Thecodiplosis japonensis*). a, miniature magnets; b, reflector (a black oval slice of plastic); c, mill arm (two copper threads); d, electrical cable.

A flight mill system was used to measure the flight capacity of *T. japonensis* females of different ages at different ambient temperatures, following the methods of Lu et al. (2019). The system consisted of a sensor and data acquisition board, a mill, and computer software. In total, 26 individual flight mills were linked to a recorder, which was connected to a computer. Each flight mill was composed of a 10-cm copper thread placed between two small magnets (Fig. 1). One anesthetized adult was attached to the lower surface of the copper thread with a droplet of 502 cyanoacrylate adhesive super glue (produced by Jinshun Adhesive Co., Ltd, Xiushui County, Jiujiang City, Jiangxi province, China) applied quickly to the pronotum. After the adult was attached, the direction of the copper thread was adjusted in order to keep the flying direction of the insect perpendicular to the copper thread. For each gall midge, the times of flight initiation and cessation and the number of mill revolutions in consecutive 5-s intervals were recorded. Flights interrupted by a >1-min interval were considered separate flights. The number of mill revolutions over a given period was used to compute flight distance, speed, and duration for each gall midge. All trials were performed in a climate-controlled chamber in VI-19.

In the first trial, the effect of age in hours on the flight performance of *T. japonensis* adults was characterized. Flight was recorded at 2, 6, and 10 h after adult emergence, with the ages of tested individuals being 0–2, 2–6, and 6–10 h old, respectively. For each hour age group, 25 adults were tested. The tests were conducted for 3 h at 26°C, 70% RH, and with a 16:8 (L:D) h photoperiod.

In the second trial, the effect of temperature on *T. japonensis* flight capacity was examined by using 2- to 6-h-old adults at 17, 21, 2, and 30°C. For each treatment, 25 adults were tested. The tests were conducted for 24 h (based on the results of adults longevity) under the same conditions as in the first trial.

### Free-Flight Assay

**Fig. 2.**
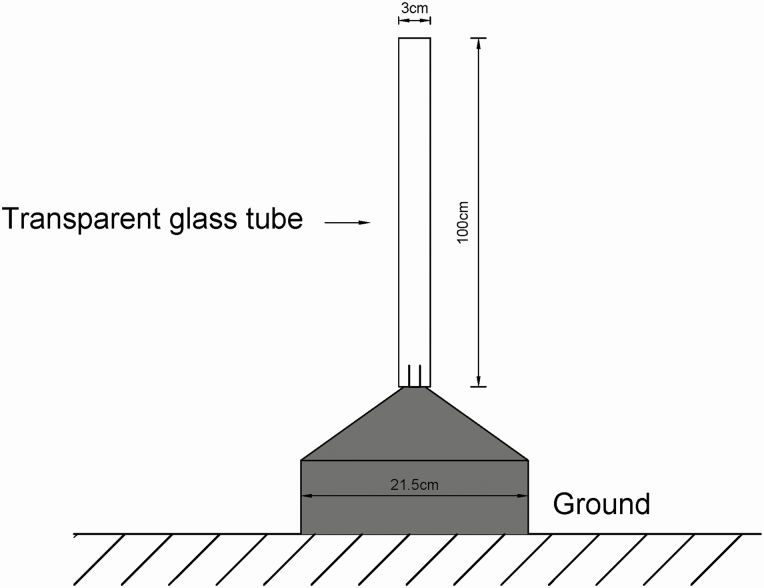
Schematic of an emergence trap used to collect newly emerged adult pine needle gall midges (*Thecodiplosis japonensis*).

The free-flight performance of newly emerging *T. japonensis* adults was surveyed by using three emergence traps. A trap was composed of a round, dark plastic basin (21.5 cm in diameter) topped by a clear plastic cylinder (diameter, 3 cm; length, 100 cm) whose top was covered with fine-mesh gauze (Fig. 2). The distance of each successive flight from takeoff to landing was recorded. In total, 30 individuals were tested and the tests were conducted at 26°C, 70% RH, and under completely enclosed conditions.

### Ability of Larvae to Move

Before observations, a digital video camera (Sony FDR-AX100E, made in Shanghai, China) with the lens facing downward was set on top of coordinate paper using a tripod. Larvae with a little water (approximately 0.05 ml) on their bodies were placed in the center of the view range, and their activity tracks were recorded onto the memory card of the video camera. Observations continued until there was no larval movement in 1 h. The vertical distance between the camera lens and the graph paper was 0.45 m, and the observation area was 125 cm^2^ (9 cm in length, 14 cm in width). Uninterrupted power was supplied during the experiment. Data in the memory card were analyzed manually on the computer, including distance moved, time, and duration for each larva. In total, 30 individuals were tested and the tests were conducted at 26°C, 70% RH, and with a 16:8 (L:D) h photoperiod, under completely enclosed conditions.

Another test was conducted in order to determine the height that larvae could jump. Before observations, a piece of graph paper was attached to the back of a cuboid transparent glass insect tank (5 × 3 × 10 cm). In front of the tank, a digital video camera was concentrated horizontally on the tank. Fifty larvae were placed on the bottom of the tank, in which water was sufficiently deep to soak up onto the bodies of the larvae. The activity tracks of each larva were recorded using the video camera. Observations continued until no larvae jumped in 1 h. The horizontal distance between the camera lens and the graph paper was 0.45 m, and the observation area was 190 cm^2^ (19 cm in width, 10 cm in height). The jumping height and timings of 30 individuals were analyzed manually on a computer. The test conditions were the same as those given above.

### Statistical Analyses

Statistical analyses were performed using SPSS (v 19.0) (Corp IBM 2010). Means of longevity of adults and flight parameters under different temperatures and ages of the insect were analyzed by one-way ANOVA, followed by multiple comparisons using LSD to identify where the differences lay. Although we used a very fine copper thread (φ < 05 mm) and a small amount of glue to stick insects, the appendages undoubtedly had an adverse effect on the flight behavior of the gall midge. To correct for this problem, when the cumulative flight distance of a single insect was <7 m (the common flight distance of females observed in a forest), it was considered as unreliable data and omitted.

## Results

### Longevity of Adults

**Fig. 3.**
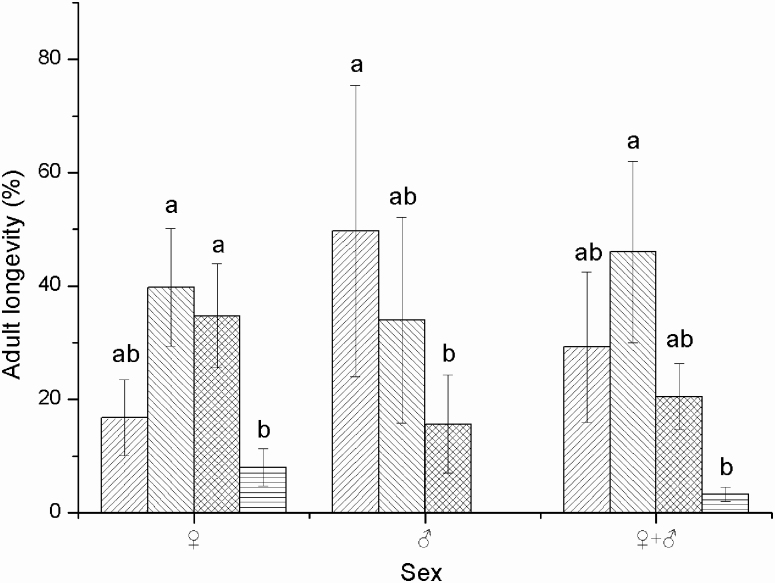
Longevity (%) of female and male *Thecodiplosis japonensis* adults in days. Forward-slashed columns show the percentage of adults whose longevity was <1 d; backslash-filled columns show the percentage of adults whose longevity was 1 d > 2 d; grid line-filled columns show the percentage of adults whose longevity was 2 d > 3 d; horizontal line-filled columns show the percentage of adults whose longevity was >3 d. Those with different lowercase letters above the error bars show significant difference among these treatments within a group (*P* < 0.05), while those with the same lowercase letters show no significant difference among these treatments (*P* > 0.05). Values are the mean ± standard deviation. Error bars indicate the standard deviation of means. *n* = 167 for male adults and 100 for female adults.

The longevity of females was 32.13 ± 2.92 h (mean ± SD) and that of males was 22.80 ± 1.70 h (mean ± SD), with females living significantly longer than males (*F* = 7.660; df = 1, 58; *P* = 0.008). Of the females that emerged on the same day, 16.75% survived for <1 d, whereas 39.75% survived for 1–2 d (Fig. 3). The longevity of approximately half the males was <1 d, whereas 34% survived for 1–2 d (Fig. 3). Of all adults (females + males), 23.75% lived longer than 2 d (Fig. 3).

### Effect of Different Temperatures on the Flying Ability

In the following analysis of the effect of temperature on the flying ability of the pine needle gall midge, reliable data were used from 11 tests at 17°C, 10 at 21°C, 10 at 26°C, and 10 at 30°C.

**Fig. 4.**
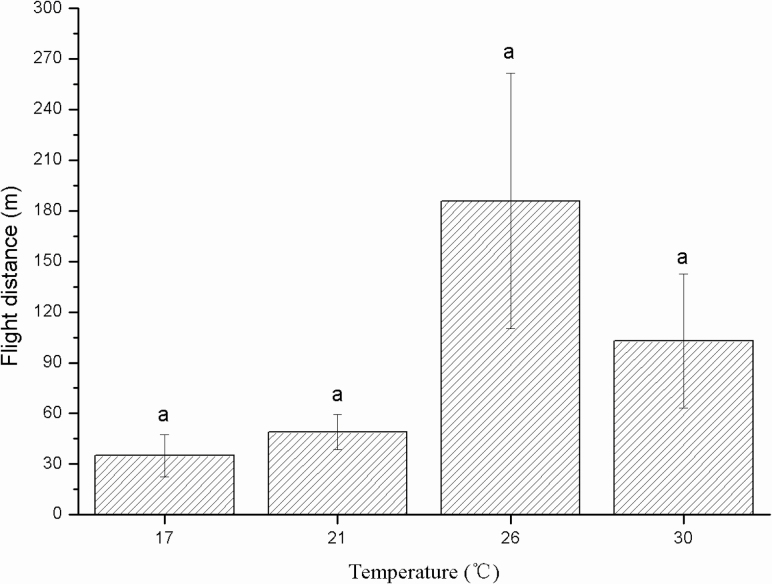
Flight distance (m) of female *Thecodiplosis japonensis* at different temperatures (°C). The same lowercase letters above the error bars indicate no significant differences among temperatures (*P* > 0.05). Values are the mean ± SD. *n* = 11 at 17°C, 10 at 21°C, 10 at 26°C, and 10 at 30°C.

The average flight distance of 6-h-old females was 34.89 m at 17°C, 48.98 m at 21°C, 185.86 m at 26°C, and 102.86 m at 30°C (Fig. 4). However, there was no significant difference in flight distance among the four temperatures (*F* = 2.598; df = 3, 37; *P* = 0.067).

**Fig. 5.**
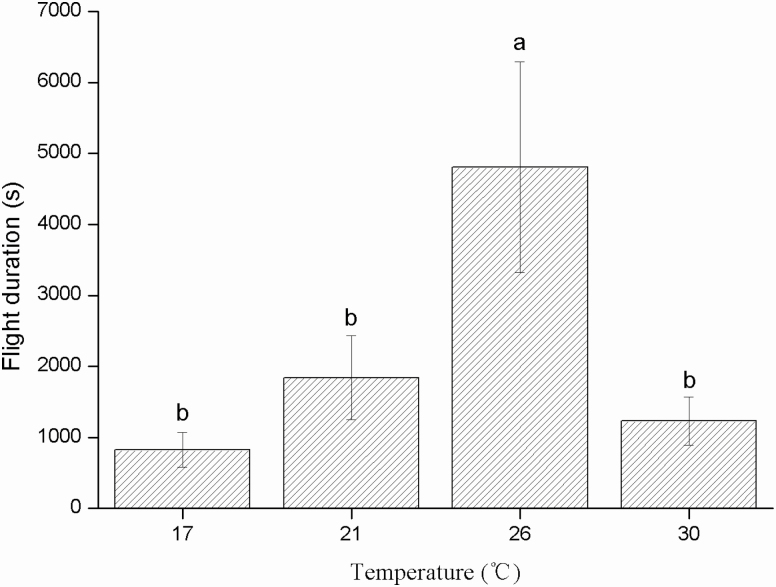
Flight duration (s) of female *Thecodiplosis japonensis* at different temperatures (°C). Different lowercase letters above the error bars indicate significant differences among temperatures (*P* < 0.05). Values are the mean ± SD. *n* = 11 at 17°C, 10 at 21°C, 10 at 26°C, and 10 at 30°C.

The flight duration of the pine needle gall midge was significantly different at the four temperatures (*F* = 4.964; df = 3, 37; *P* = 0.005). At 26°C, the average flight duration was 4,806.26 s; the duration was significantly lower at 17°C (824.74 s; *P* = 0.001), 21°C (1,840.38 s; *P* = 0.014), and 30°C (1,230.03 s; *P* = 0.004) (Fig. 5).

**Fig. 6.**
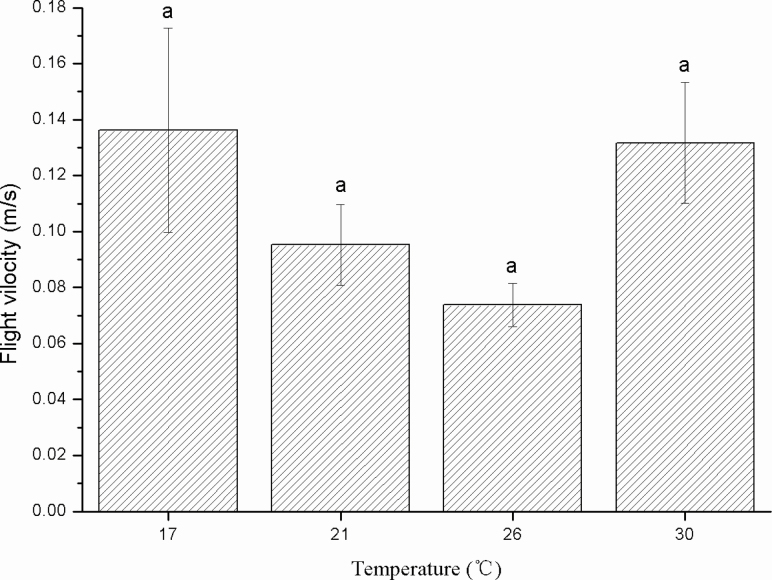
Flight velocity (m/s) of female *Thecodiplosis japonensis* at different temperatures (°C). The same lowercase letters above the error bars indicate no significant differences among temperatures (*P* > 0.05). Values are the mean ± SD. *n* = 11 at 17°C, 10 at 21°C, 10 at 26°C, and 10 at 30°C.

The flight speed was not significantly different at the different temperatures (*F* = 1.592; df = 3, 37; *P* = 0.208). The mean flight speed was 0.11 m·s^−1^ in 3 h of tethered flight (Fig. 6).

### Effect of Age on Flight Capacity

For each age group of females (0–2, 2–6, and 6–10 h), reliable data from 10 tests were used in the following analysis.

Among the different ages of the pine needle gall midge, there were no significant differences in flight distance (*F* = 0.18; df = 2, 27; *P* = 0.835), duration (*F* = 0.27; df = 2, 27; *P* = 0.767), or speed (*F* = 0.80; df = 2, 27; *P* = 0.462) (Table 1). The mean flight distance was 192.71 m in 24 h of tethered flight; the mean flight duration was 2,035.48 s; and the mean flight speed was 0.16 m·s^−1^. The maximum flight distance was 667.59 m; the longest flight time was 6,222.34 s; and the fastest flight speed was 0.44 m·s^−1^.

**Table 1.** Flight performance (mean ± SD) of female *Thecodiplosis japonensis* at different ages (hours) during 24 h of flight

Ages (hours)	Flight distance (m)	Flight velocity (m/s)	Flight duration (s)
2	219.31 ± 71.87a	0.16 ± 0.02a	2,337.69 ± 581.35a
6	190.66 ± 57.38a	0.19 ± 0.03a	1,761.81 ± 501.73a
10	168.14 ± 49.47a	0.14 ± 0.02a	2,006.93 ± 586.52a

*n* = 10 for each age. The same lowercase letters for each flight characteristic indicate no significant differences among ages (*P* > 0.05).

### Free-Flight Performance

Sixteen free flights of newly emerged adults were observed. The longest free-flight distance, from the point of takeoff to the point of landing, was 60.90 cm. In a distance of 1 m, *T. japonensis* adults took off an average of 4.88 times.

### Movement Ability of Larvae

In this test, without moisture on them, larvae moved only a very short distance by rolling over. However, when their body surface was wet, larvae would crawl or jump, and the maximum crawling distance within 5 min was 4.3 cm. The average height of a jump was 1.45 ± 0.18 cm (mean ± SD), and the average distance jumped was 4.17 ± 0.39 cm. The highest jump was 5.7 cm, the farthest jump was 9.6 cm, and the farthest distance moved was 27.13 cm.

## Discussion

In this study, more than 80% of females and more than 50% of males of *T. japonensis* lived longer than 1 d, which is in contrast to the conclusion of Soné (1987) who reported that the life span of males and females was typically only 1 d in Kyoto, Japan. This difference in longevity might be due to different test conditions. In this study, the results were obtained through field observations, and unfortunately, the experimental conditions of Soné (1987) were not considered. However, in preliminary laboratory observations in the laboratory (25°C, 60% RH), the life span of *T. japonensis* adults was less than 24 h (unpublished).

In the flight mill experiments, the fastest flying speed for *T. japonensis* was 0.44 m·s^−1^ (26.4 m·min^−1^), which is relatively slow compared with other dipterans. For example, the flight speed is 67.2 m·min^−1^ for *Aedes gambiae* Giles and 68.4 m·min^−1^ for *Culex thalassius* Theobald (Culicidae) (Yukawa et al. 2019). Based on the fastest flight speed and the longest flight time (6,222.34 s), the theoretical flight distance of the pine needle gall midge could reach 2.74 km within 24 h. Because *T. japonensis* is univoltine, this distance could also be regarded as the maximum that adults could achieve by actively flight in a year. In the current study, the maximum flight distance of 2- to 10-h-old adults was 667.59 m, which is greater than the estimation (up to 400 m) of the dispersal capacity of *T. japonensis* adults by Lee et al. (1997). Lee et al. (2007) analyzed the dispersal process of *T. japonensis* using a lattice model, and showed the range expanded approximately 8.2 km/year in a Mokpo population and 5.2 km/year in a Seoul population, during the range expansion phase. When considering the speeds of range expansion mentioned above, human-mediated movement and wind may assist the long-distance range expansion of *T. japonensis*. Cases of human-mediated movement of *T. japonensis* were reported in 1974, 1981, 1982, 1984, and 1990 (Choi and Park 2012).

Insects partition their effort between lift and thrust in flight mill systems, which is different from those in free flight (Riley et al. 1997). Taylor et al. (2010) also found that greater flight activity and flight distances may occur when insects are allowed to rest and consume water and/or feed in natural settings. In this study, free-flight tests showed that *T. japonensis* adults were likely to take a rest during a flight. In addition, the adults were not provided with food and water during the experiment. Therefore, the dispersal capacity of *T. japonensis* adults might have been underestimated in this study.

Many studies show that insect flight behavior is closely related to temperature (Duan et al. 1998, Jiang et al. 2002, Doane and Olfert 2008, Lu et al. 2019). The optimum temperature for flight of *Sitobion avenae* is 12–22°C (Cheng et al. 2002). It is difficult for this aphid to take off at temperatures below 8°C, and if flight is achieved then flight time is shortened considerably (Cheng et al. 2002). The flight distances of *Corythucha ciliata* are significantly different at different temperatures, with the strongest flight capacity at 25°C (Lu et al. 2019). The best temperature for flight of *Sitodiplosis mosellana* is similar to the normal field conditions when adults emerge (Doane and Olfert 2008). The flight behavior of *T. japonensis* adults was also affected by temperature, and they were most active at 26°C, with the average flight distance and duration both longer than those at 17, 21, and 30°C. This optimum temperature for flight is slightly higher than the optimum temperatures of overwintered larvae (22.3°C) and pupae (24.0°C) (Choi and Park 2012).

Movement also varies depending on insect age (Rudd and McEvoy 1996). One-day-old diamond back moths (*Plutella xylostella*) are the weakest fliers, whereas 3-d-old moths are strongest, with 10, 546 m the longest flight distance (Wei et al. 2013). The flight activity of 1- and 2-d-old beet webworms (*Loxostege sticticalis*) is significantly less than that of 4- and 5-d-old moths (Tang et al. 2016). However, age within 12 h did not significantly affect the flight performance of *T. japonensis* in this study. This absence of an age effect may be an adaptation to the short life span of *T. japonensis* (more than 75% of adults survived <2 d; Fig. 3).

Mature *T. japonensis* larvae leave the galls and drop to the ground from November to March of the following year (CABI 2019). These larvae crawl into the litter layer or the surface soil, where most spin cocoons (Ko 1982). According to Hyun (1982), soil moisture is the most important factor influencing the mortality of larvae hibernating in the soil. In the current study, moisture was also the key factor that determined larval movement. However, the distance that larvae moved was determined on a smooth surface in this study, and therefore, the influence of the soil environment on the movement of larvae needs to be studied further.

### Conclusions

The longevity of pine needle gall midge adults was relatively short, and only 23.75% of adults lived longer than 2 d, with the longest life spans less than 4 d. Because *T. japonensis* is a univoltine insect, the short life span limits the active dispersal distance. In addition, the environmental temperature was also an important factor limiting adult flight behavior. Female flight distance and duration at 26°C were significantly longer than those at 17°C and 21°C. The age of *T. japonensis* had no significant effect on flight distance, duration, or speed. The active dispersal of larvae that relied on crawling and jumping was greater than that of larvae that only rolled over. A primary measure to control the pine needle gall midge could be the timely springtime treatment of soil that is near attacked host trees.

## References

[CIT0001] Baguette M, Stevens V M, and Clobert J. 2014 The pros and cons of applying the movement ecology paradigm for studying animal dispersal. Mov. Ecol. 2: 13.

[CIT0002] CABI 2019 Thecodiplosis japonensis. *In* Invasive species compendium. CAB International, Wallingford, United Kingdom www.cabi.org/isc.

[CIT0003] Cheng D, Tian Z, Li H, Sun J, and Chen J. 2002 Effects of temperature and humidity on flight ability of *Sitobion avenae* [J]. Acta Entomol. 1: 80–85.

[CIT0004] Choi W, and Park Y-S. 2012 Dispersal patterns of exotic forest pests in South Korea. Insect Sci. 19: 535–548.

[CIT0005] Choi W I, Ryoo M I, Chung Y J, and Park Y S. 2011 Geographical variation in the population dynamics of *Thecodiplosis japonensis*: causes and effects on spatial synchrony. Popul. Ecol. 53: 429–439.

[CIT0006] Corp IBM 2010 IBM SPSS Statistics for Windows, version 19.0. IBM Corp., Armonk, NY.

[CIT0007] Doane J, and Olfert O. 2008 Seasonal development of wheat midge, *Sitodiplosis mosellana* (Géhin) (Diptera: Cecidomyiidae), in Saskatchewan, Canada. Crop Prot. 27: 951–958.

[CIT0008] Duan J J, Weber D C, and Dorn S. 1998 Flight behavior of pre- and postdiapause apple blossom weevils in relation to ambient temperature. Entomol. Exp. Appl. 88: 97–99.

[CIT0009] Hyun J S 1982 The ecology of the pine needle gall midge (*Thecodiplosis japonensis* Uchida et Inouye) and its control strategies. Korean J. Plant Prot. 21: 163–166.

[CIT0010] Jiang X, Luo L, Li K, Cao Y, Hu Y, and Liu Y. 2002 Influence of temperature on flight capacity of the beet armyworm, *Spodoptera exigua*. Acta Entomol. Sin. 45: 275–278.

[CIT0011] Ko J H 1982 The present status of damage and control of pine gall midge in Korea. Korean J. Plant Prot. 21: 159–162.

[CIT0012] Ko J, and Lee B. 1975 Influence of the wind on the dispersion of the pine gall-midge (*Thecodiplosis japonensis*) -tested in the wind tunnel. Korean J. Entomol. 5: 13–16.

[CIT0013] Kwon S, Chung K and Ryu J. 1978 Radioisotope labelling method of pine gall midge (*Thecodiplosis japonensis* Uchida Et Inouye). Korean J. Plant Prot. 17: 155–160.

[CIT0014] Lee B, and Lee S. 1994 Status of the pine needle gall midge in Korea. *In* Ecology and evolution of gall-forming insects [Krasnoyarsk, Siberia, August. 9–13, 1993]. 37–41.

[CIT0015] Lee B Y, Chung Y J, Park K N, Byun B H, and Bae W I. 1997 Distribution of pine needle gall midge, *Thecodiplosis japonensis* Uchida et Inouye (Diptera: Cecidomyiidae), infestations in Korea: a brief history. FRI J. For. Sci. 56: 13–20.

[CIT0016] Lee S D, Park S, Park Y S, Chung Y J, Lee B Y, and Chon T S. 2007 Range expansion of forest pest populations by using the lattice model. Ecol. Model. 203: 157–166.

[CIT0017] Li H, Zhao J, Zhou C, Merkle S A, and Zhang J-F. 2015 Morphologic characters and element content during development of *Pinus tabuliformis* seeds. J. For. Res. 27: 67–74.

[CIT0018] Lu S, Wei M, Yuan G, Cui J, and Gong D. 2019 Flight behavior of the sycamore lace bug, *Corythucha ciliata*, in relation to temperature, age, and sex. J. Integr. Agric. 1: 2330–2337.

[CIT0019] Luo Y 1993 Introduction to main forest diseases and insect pests in South Korea. Forest Pest and Disease. 4: 39–42.

[CIT0020] Mazzi D, and Dorn S. 2012 Movement of insect pests in agricultural landscapes. Ann. Appl. Biol. 160: 97–113

[CIT0021] McManus M L 1988 Weather, behaviour and insect dispersal. Mem. Entomol. Soc. Can. 120: 71–94.

[CIT0022] Nam Y, and Choi W I. 2014 An empirical predictive model for the spring emergence of *Thecodiplosis japonensis* (Diptera: Cecidomyiidae): model construction and validation on the basis of 25 years of field observation data. J. Econ. Entomol. 107: 1136–1141. 25026674 10.1603/ec14073

[CIT0023] Nathan R, Getz W M, Revilla E, Holyoak M, Kadmon R, Saltz D, and Smouse P E. 2008 A movement ecology paradigm for unifying organismal movement research. Proc. Natl. Acad. Sci. USA. 105: 19052–19059. 19060196 10.1073/pnas.0800375105 PMC2614714

[CIT0024] Orr D B, Salazar C G, and Landis D A. 2000 *Trichogramma* nontarget impacts: a method for biological control risk assessment, pp. 111–125. *In* Follett P A and Duan J J (eds.), Nontarget effects of biological control. Kluwer Academic Publishers, Boston, MA.

[CIT0025] Park Y S, and Chung Y J. 2006 Hazard rating of pine trees from a forest insect pest using artificial neural networks. For. Ecol. Manag. 222: 222–233.

[CIT0026] Park K, Miura T, and Hirashima Y. 1985 Outbreaks history and present status of the pine needle gall midge in Korea. Esakia. 23: 115–118.

[CIT0027] Rafter M A, McCulloch G A, Daglish G J, Gurdasani K, and Walter G H. 2018 Polyandry, genetic diversity and fecundity of emigrating beetles: understanding new foci of infestation and selection. J. Pest Sci. 91: 287–298.

[CIT0028] Ren X, Li X, Wang J, Tan B, Wang L, and Zhang Y. 2018 Allelopathic effect of black pine (*Pinus thunbergii*) needles and litter on four cover plants. Agric. Biotech. 7: 156–159.

[CIT0029] Riley J R, Downham M C A, and Cooter R J. 1997 Comparison of the performance of *Cicadulina* leafhoppers on flight mills with that to be expected in free. Entomol. Exp. Appl. 83: 317–322.

[CIT0030] Roderick G K, and Caldwell R L. 1992 An entomological perspective on animal dispersal, pp. 274–290. *In* Stenseth N C and Lidicker W Z (eds.), Animal dispersal. Springer, Dordrecht, The Netherlands.

[CIT0031] Rudd N T, and McEvoy P B. 1996 Local dispersal by the cinnabar moth *Tyria jacobaeae*. Ecol. Appl. 6: 285–297.

[CIT0032] Schellhorn N, Bianchi F and Hsu C. 2014 Movement of entomophagous arthropods in agricultural landscapes: links to pest suppression. Ann. Rev. Entomol. 59: 559–581. 24397523 10.1146/annurev-ento-011613-161952

[CIT0033] Smith S M 1996 Biological control with *Trichogramma*: advances, successes, and potential of their use. Annu. Rev. Entomol. 41: 375–406. 15012334 10.1146/annurev.en.41.010196.002111

[CIT0034] Soné K 1986 Mortality factors before gall formation by the pine needle gall midge, *Thecodiplosis japonensis* Uchida Et Inouye (Diptera: Cecidomyiidae). J. Jpn. For. Soc. 68: 32–34.

[CIT0035] Soné K 1987 Population dynamics of the pine needle gall midge, *Thecodiplosis japonensis* Uchida Et Inouye (Diptera, Cecidomyiidae). J. Appl. Entomol. 103: 386–402.

[CIT0036] Tang J, Cheng Y, Luo L, Jiang X, and Zhang L. 2016 Effects of age, temperature and relative humidity on free flight activity of the beet webworm *Loxostege sticticalis*. Plant Prot. 42: 79–83.

[CIT0037] Taylor L 1963 Analysis of the effect of temperature on insects in flight. J. Anim. Ecol. 1: 99–117.

[CIT0038] Taylor R A J, Bauer L S, Poland T M, and Windell K N. 2010 Flight performance of *Agrilus planipennis* (Coleoptera: Buprestidae) on a flight mill and in free flight. J. Insect Behav. 23: 128–148.

[CIT0039] Turchin P 1998 Quantitative analysis of movement: measuring and modeling population redistribution in animals and plants. Sinauer Associates, Sunderland, MA.

[CIT0040] Walters K, and Dixon A. 1984 The effect of temperature and wind on the flight activity of cereal aphids. Ann. Appl. Biol. 104: 17–26.

[CIT0041] Wei S, Fan X, Gu Y, Wang Z, Gong Y, Jin G, and Shi B. 2013 Preliminary study of the effect of age and mating on the flight ability of the diamondback moth *Plutella xylostella*. Chin. J. Appl. Entomol. 50: 474–482.

[CIT0042] Yukawa J, Moriya T, and Kanmiya K. 2019 Comparison in the flight ability between the soybean pod gall midge, *Asphondylia yushimai* and the aucuba fruit gall midge, *A. aucubae* (Diptera: Cecidomyiidae)[J]. Appl. Entomol. Zool. 54: 167–174.

